# Electronic and optical properties of heterostructures based on transition metal dichalcogenides and graphene-like zinc oxide

**DOI:** 10.1038/s41598-018-30614-3

**Published:** 2018-08-13

**Authors:** Sake Wang, Hongyu Tian, Chongdan Ren, Jin Yu, Minglei Sun

**Affiliations:** 1 College of Science, Jinling Institute of Technology, Nanjing, Jiangsu 211169 China; 2 School of Physics and Electronic Engineering, Linyi University, Linyi, Shandong 276005 China; 3 Department of Physics, Zunyi Normal College, Zunyi, Guizhou 563002 China; 4 School of Materials Science and Engineering, Southeast University, Nanjing, Jiangsu 211189 China; 5 School of Mechanical Engineering, Southeast University, Nanjing, Jiangsu 211189 China; 6 Institute of High Performance Computing, A*STAR, Singapore, 138632 Singapore

## Abstract

The structural, electronic, and optical properties of heterostructures formed by transition metal dichalcogenides MX_2_ (M = Mo, W; X = S, Se) and graphene-like zinc oxide (ZnO) were investigated using first-principles calculations. The interlayer interaction in all heterostructures was characterized by van der Waals forces. Type-II band alignment occurs at the MoS_2_/ZnO and WS_2_/ZnO interfaces, together with the large built-in electric field across the interface, suggesting effective photogenerated-charge separation. Meanwhile, type-I band alignment occurs at the MoSe_2_/ZnO and WSe_2_/ZnO interfaces. Moreover, all heterostructures exhibit excellent optical absorption in the visible and infrared regions, which is vital for optical applications.

## Introduction

Recently, transition-metal dichalcogenides (TMDs) have attracted much attention because of their interesting electronic^1,2^, mechanical^3^, thermal^4,5^, and optical^6^ properties. Their monolayers can be prepared by either mechanical exfoliation or chemical growth^7^. Investigations on the application of TMDs in nanoelectronics^8,9^, catalysis^10,11^, spintronics^12,13^, and valleytronics^14,15^ indicate that TMDs are a category of very promising two-dimensional (2D) materials.

**Figure 1:**
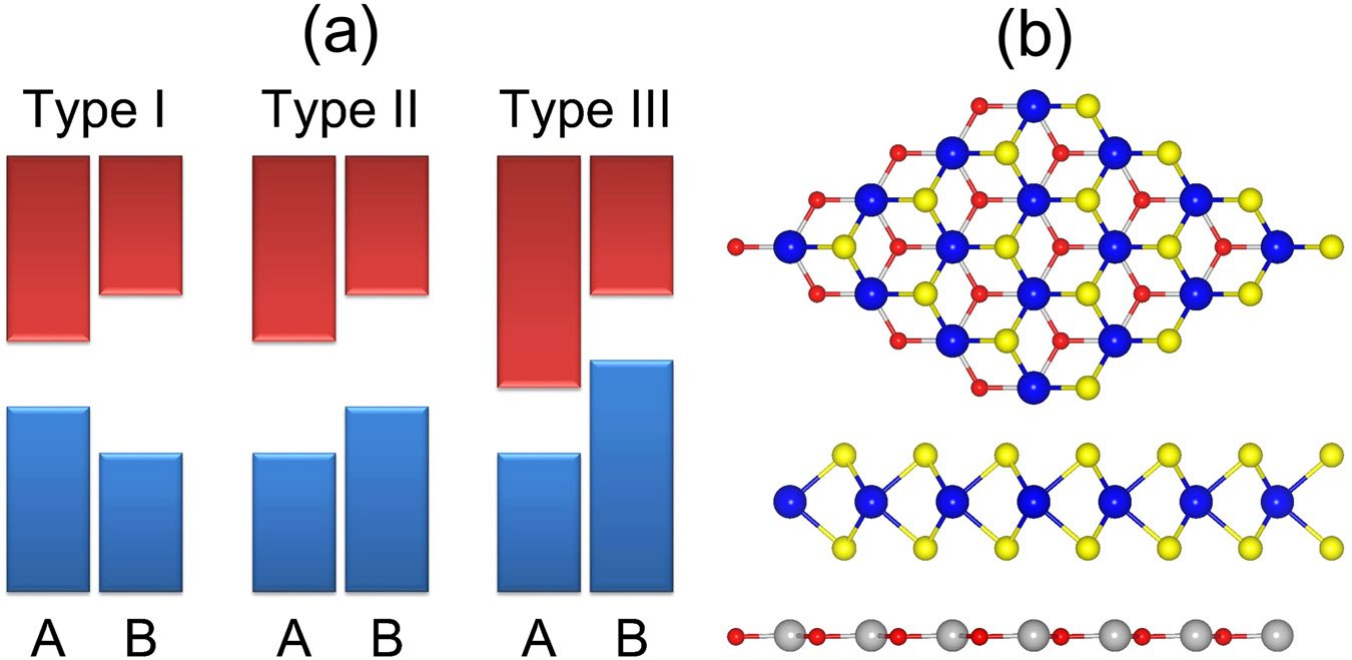
(**a**) Schematic presentation of type-I, type-II, and type-III heterostructures; the conduction bands are shown in red, and the valence bands are shown in blue. (**b**) Schematic illustration of crystal structures of the MX_2_/ZnO heterostructure; the red, yellow, grey, and blue spheres represent M, X, Zn, and O atoms, respectively.

At the same time, the formation of 2D van der Waals (vdW) heterostructures has been widely adopted to tune the properties of 2D materials. The vdW heterostructure has been extensively investigated in theoretical and experimental studies^16–28^, and the band alignments at the interface of a semiconducting vdW heterostructure are found to be vital for its applications. Heterostructures can be divided into three types according to the band alignment: type I (symmetric), type II (staggered), or type III (broken)^29^, as shown in Fig. 1(a). In a type-I heterostructure, the conduction band minimum (CBM) and the valence band maximum (VBM) of two composite layers (A and B) obey the following rule: VBM_B_ < VBM_A_ < CBM_A_ < CBM_B_. Since the VBM and CBM of a type-I heterostructure are located in one layer, efficient recombination of the photogenerated electrons and holes can occur when it is irradiated by light. Therefore, type-I heterostructures have been widely used in optical devices such as light-emitting diodes (LEDs)^30^. In contrast, in a type-II heterostructure, the CBM and the VBM of two composite layers (A and B) obey the following rule: VBM_A_ < VBM_B_ < CBM_A_ < CBM_B_. Therefore, the VBM and CBM of a type-II heterostructure exist in different layers. Since the photogenerated electron–hole pairs can be split at the interface, with electrons transferred to one layer and the holes to the other, this heterostructure has been demonstrated to be a fundamental component of photovoltaic devices. For example, Bernardi *et al*.^31^ investigated the photovoltaic devices based on a MoS_2_/WS_2_ bilayer, they revealed that the MoS_2_ and WS_2_ monolayers form a type-II heterostructure, which can yield a power-conversion efficiency of up to ∼1% and much higher power densities than existing ultrathin solar cells. On the other hand, in a type-III heterostructure, the CBM and the VBM of two composite layers (A and B) obey the following rule: VBM_A_ < CBM_A_ < VBM_B_ < CBM_B_. Type-III heterostructures have been successfully applied in tunnelling field-effect transistors^32^. As described earlier, in addition to being an interesting research topic, the formation of vdW heterostructures by TMDs and other materials can widen the application of TMDs.

Graphene-like ZnO (ZnO) is also a widely investigated 2D semiconducting material^33–36^. Moreover, ZnO layers have been experimentally synthesized^37–39^. In the study reported here, we designed four different MX_2_/ZnO heterostructures: MoS_2_/ZnO, WS_2_/ZnO, MoSe_2_/ZnO, and WSe_2_/ZnO. The structural, electronic, and optical properties of these heterostructures were investigated.

The lattice parameters of MoS_2_, WS_2_, MoSe_2_, WSe_2_, and ZnO are 3.16, 3.17, 3.29, 3.29, and 3.29 Å, respectively. The bandgaps of MoS_2_, MoSe_2_, WS_2_, WSe_2_ and ZnO are 2.24, 2.37, 1.99, 2.12 and 3.29 eV respectively, and they are all direct-bandgap semiconductors. In an earlier study, Defo *et al*.^40^ demonstrated that the electronic properties of MX_2_ are rather sensitive to strain. Therefore, we chose to vary the lattice constant of ZnO and fix the lattice constants of MX_2_ for constructing the heterostructures. The interlayer lattice mismatches in the MoS_2_/ZnO and WS_2_/ZnO heterostructures are 4.11% and 3.79% respectively, which are quite small. For each MX_2_/ZnO heterostructure, several possible stacking patterns were examined (Fig. S1 in Supporting Information). Interestingly, all of the heterostructures favour the same stacking pattern, as shown in Fig. 1(b). The binding energy is defined as ${E}_{{\rm{b}}}={E}_{{{\rm{MX}}}_{{\rm{2}}}}+{E}_{{\rm{ZnO}}}-{E}_{{{\rm{MX}}}_{{\rm{2}}}/{\rm{ZnO}}}$, where ${E}_{{{\rm{MX}}}_{{\rm{2}}}}$, *E*_ZnO_, and ${E}_{{{\rm{MX}}}_{{\rm{2}}}/{\rm{ZnO}}}$ are the total energy of MX_2_, ZnO, and the MX_2_/ZnO heterostructure, respectively. The binding energy of the MoS_2_/ZnO, WS_2_/ZnO, MoSe_2_/ZnO and WSe_2_/ZnO heterostructures are 269, 264, 285 and 282 meV respectively, while the corresponding interlayer distances are 2.91, 2.98, 2.89 and 2.89 Å respectively, indicating the typical vdW nature of the interaction between the two layers.

**Figure 2:**
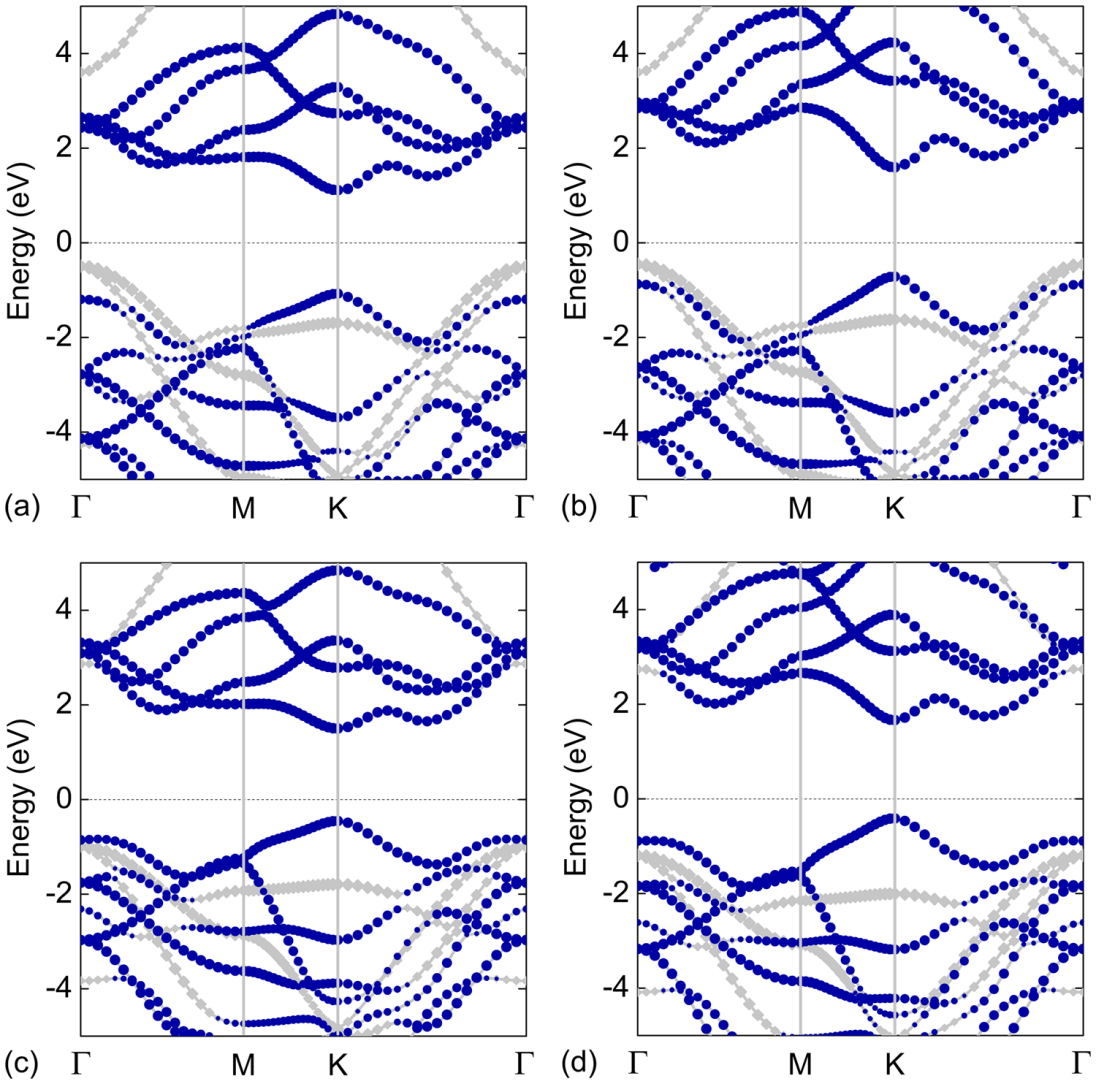
Projected band structures of the MoS_2_/ZnO, WS_2_/ZnO, MoSe_2_/ZnO, and WSe_2_/ZnO vdW heterostructures; the royal and grey symbols represent the contribution of MX_2_ and ZnO, respectively; the Fermi level is set to zero, and it is indicated by the black dashed line.

The projected band structures of the MoS_2_/ZnO, WS_2_/ZnO, MoSe_2_/ZnO, and WSe_2_/ZnO vdW heterostructures are shown in Fig. 2. These heterostructures can be divided into two categories. The first category includes the MoS_2_/ZnO and WS_2_/ZnO heterostructures, both of which have a type-II band alignment. They are semiconductors with indirect bandgaps of 1.60 and 2.05 eV, respectively. The CBM and VBM of the MoS_2_/ZnO (or WS_2_/ZnO) heterostructure are predominately contributed by the MoS_2_ (or WS_2_) and ZnO layers respectively. The second category includes the MoSe_2_/ZnO and WSe_2_/ZnO heterostructures, both of which have a type-I band alignment. Both the CBM and VBM of MoSe_2_/ZnO and WSe_2_/ZnO heterostructures are located at the *K* point in BZ, which suggest that they are direct-bandgap semiconductors. The bandgaps of MoSe_2_/ZnO and WSe_2_/ZnO heterostructures are 1.96 and 2.08 eV, respectively. Moreover, both the CBM and VBM of these two heterostructures originate from the TMD layer.

**Figure 3:**
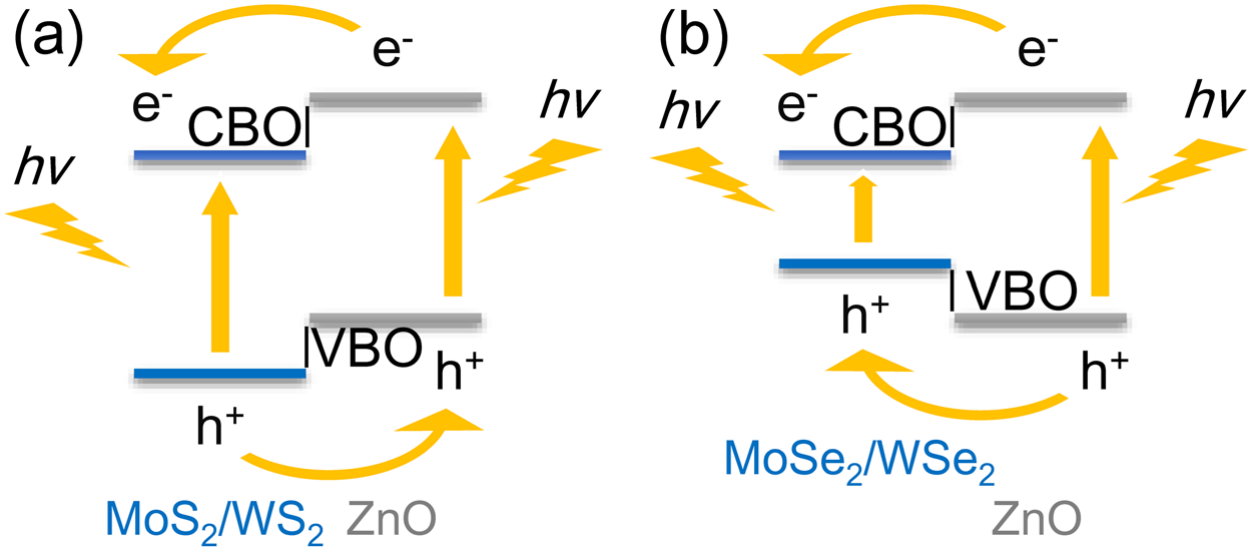
Schematic illustration of the migration of photogenerated electrons and holes at the (**a**) MoS_2_/ZnO and WS_2_/ZnO interfaces; and the (**b**) MoSe_2_/ZnO and WSe_2_/ZnO interfaces.

Previously, many reports^41–43^ suggested that MoS_2_ and WS_2_ have the potential for application in photocatalysts for water splitting. The main obstacle to obtain a high-efficiency photocatalyst is the problem of electron–hole recombination. In the MoS_2_/ZnO and WS_2_/ZnO heterostructures, the conduction-band offset (CBO) and valence-band offset (VBO) between the MoS_2_ (or WS_2_) and ZnO layers are approximately 2.49 (or 2.00) and 0.58 (or 0.26) eV respectively, as shown in Fig. 3(a). Driven by the CBO, the photogenerated electrons in ZnO tend to move to the CB of the MoS_2_ (or WS_2_) layer, while the photogenerated holes in the MoS_2_ (or WS_2_) layer are readily migrate to the VB of the ZnO layer with the assistance of the VBO. Therefore, the problem of electron–hole recombination can be overcome with these band offsets.

**Figure 4:**
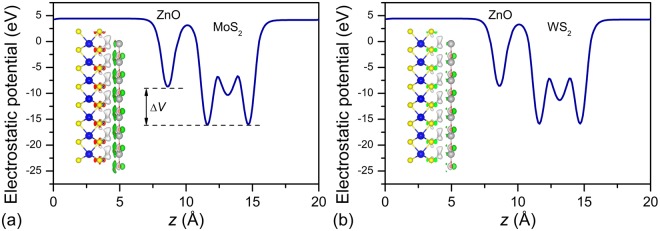
Potential drop across the interface of the (**a**) MoS_2_/ZnO and (**b**) WS_2_/ZnO vdW heterostructure. The isosurface of charge difference (set to 0.003 e/Å^3^) is also shown in the corresponding figure; the yellow and cyan regions denote the gain and loss of electrons, respectively.

The built-in electric field plays an important role in determining the catalytic activity of a photocatalyst because a large built-in electric field can further boost the migration of photogenerated charges. The insets in Fig. 4 present the isosurfaces of charge difference of the MoS_2_/ZnO and WS_2_/ZnO vdW heterostructures. The ZnO layer always acts as a donor. The transferred charge is 0.016 (or 0.012) |e| for the MoS_2_/ZnO (or WS_2_/ZnO) vdW heterostructure according to the Bader charge-population analysis^44–46^, which can induce a large potential drop across the interface of the heterostructure, as shown in Fig. 4. The potential drop across the MoS_2_/ZnO (or WS_2_/ZnO interface) is 7.38 (or 7.33) eV, which can induce a large built-in electric field from the MoS_2_ (or WS_2_) layer to the ZnO layer, and this field should exert some effect on the photogenerated electron–hole recombination in the MoS_2_/ZnO (or WS_2_/ZnO) vdW heterostructure.

The CBO and VBO in the MoSe_2_/ZnO and WSe_2_/ZnO heterostructures also play an important role. As shown in Fig. 3(b), the CBO and VBO in the MoSe_2_/ZnO (or WSe_2_/ZnO) heterostructure are 1.37 (or 1.07) and 0.53 (or 0.78) eV, respectively. With these band offsets, both the photogenerated electrons and holes tend to move from the ZnO to the TMD layer, while the photogenerated electrons and holes in the TMD layer are prohibited from escaping. Thus, the photogenerated electrons and holes tend to recombine again, which would be a useful feature for optical devices such as LEDs^30^.

**Figure 5:**
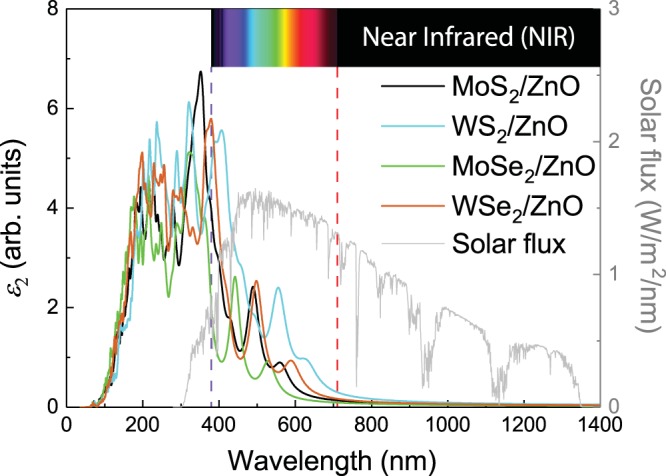
Imaginary parts of dielectric constants of the MoS_2_/ZnO, WS_2_/ZnO, MoSe_2_/ZnO, and WSe_2_/ZnO vdW heterostructures; the range of light absorption by each heterostructure overlaps the wavelength range of the incident AM1.5 G solar flux.

The imaginary parts of the dielectric functions of the MoS_2_/ZnO, WS_2_/ZnO, MoSe_2_/ZnO, and WSe_2_/ZnO vdW heterostructures are shown in Fig. 5. All the heterostructures show good ability to absorb light in the visible and near-infrared (NIR) regions, which is evident from the high absorption peaks at approximately 488, 555, 441, and 498 nm in the visible region of their respective spectra. Since the wavelengths of light arriving at the earth are mainly in the visible and NIRregions^47^, these heterostructures are promising components for various optical, photovoltaic and photocatalytic applications.

In summary, the structural, electronic, and optical properties of the MoS_2_/ZnO, WS_2_/ZnO, MoSe_2_/ZnO, and WSe_2_/ZnO vdW heterostructures were systematically investigated using first-principles calculations. The interactions at all the TMD/ZnO interfaces are dominated by vdW forces. The MoS_2_/ZnO and WS_2_/ZnO vdW heterostructures are indirect-bandgap semiconductors with bandgaps of 1.60 and 2.05 eV, respectively. The CBM is contributed by the TMD layer, while the VBM is contributed by the ZnO layer, indicating the formation of a type-II heterostructure, which can promote the separation of photogenerated electron–hole pairs. Moreover, large built-in electric fields are stabilized at both the MoS_2_/ZnO and WS_2_/ZnO interfaces, which will further separate the photogenerated charges. On the other hand, the MoSe_2_/ZnO and WSe_2_/ZnO vdW heterostructures are direct-bandgap semiconductors with bandgaps of 1.96 and 2.08 eV respectively. Both the CBM and VBM originate from the TMD layer, thus a type-I heterostructure is formed. In addition, the MoS_2_/ZnO, WS_2_/ZnO, MoSe_2_/ZnO, and WSe_2_/ZnO vdW heterostructures are all high solar-flux collectors. Therefore, these hetersotructures have great potential for application in optical, photovoltaic, and photocatalytic devices.

## Methods

First-principles calculations were carried out by using the Vienna Ab Initio Simulation Package^48^, which is based on the density functional theory (DFT) in a plane-wave basis set with the projector-augmented wave method^49^. For the exchange-correlation functional, the generalized gradient approximation of Perdew, Burke, and Ernzerhof ^50,51^ was used to obtain the geometric structures, while the Heyd–Scuseria–Ernzerhof hybrid functional^52,53^ was used to calculate the electronic and optical properties. The DFT-D3 method of Grimme^54^ was used to account for the dispersion forces. The energy cutoff for plane-wave expansion was set to 550 eV, and the first Brillouin zone was sampled by a 21 × 21 × 1 Monkhorst–Pack^55^ *k*-point grid. The thickness of the vacuum region was set to 20 Å to avoid interference between the periodic images. All the structures were fully relaxed until the Hellmann–Feynman force on each atom was <0.01 eV/Å.

## Electronic supplementary material

Supporting Information
