# Supplementary material for: Electronic and optical properties of heterostructures based on transition metal dichalcogenides and graphene-like zinc oxide
Source: Sci Rep. 2018 Aug 13;8:12009. doi: 10.1038/s41598-018-30614-3 (PMC6089903; doi:10.1038/s41598-018-30614-3)
Supplement: Supplementary file 1 — Supporting Information [file 41598_2018_30614_MOESM1_ESM.docx]

**Supporting Information**

**Electronic and optical properties of heterostructures based on transition metal dichalcogenides and graphene-like zinc oxide**

Sake Wang^1*^, Hongyu Tian^2^, Chongdan Ren^3^, Jin Yu^4^, and Minglei Sun^56*^

*^1^College of Science, Jinling Institute of Technology, Nanjing, Jiangsu 211169, China*

*^2^School of Physics and Electronic Engineering, Linyi University, Linyi, Shandong 276005, China*

*^3^Department of Physics, Zunyi Normal College, Zunyi, Guizhou 563002, China*

*^4^School of Materials Science and Engineering, Southeast University, Nanjing, Jiangsu 211189, China*

*^5^School of Mechanical Engineering, Southeast University, Nanjing, Jiangsu 211189, China*

*^6^Institute of High Performance Computing, A*STAR, Singapore 138632, Singapore*

E-mail: IsaacWang@jit.edu.cn, [mingleisun@outlook.com](mailto:mingleisun@outlook.com)


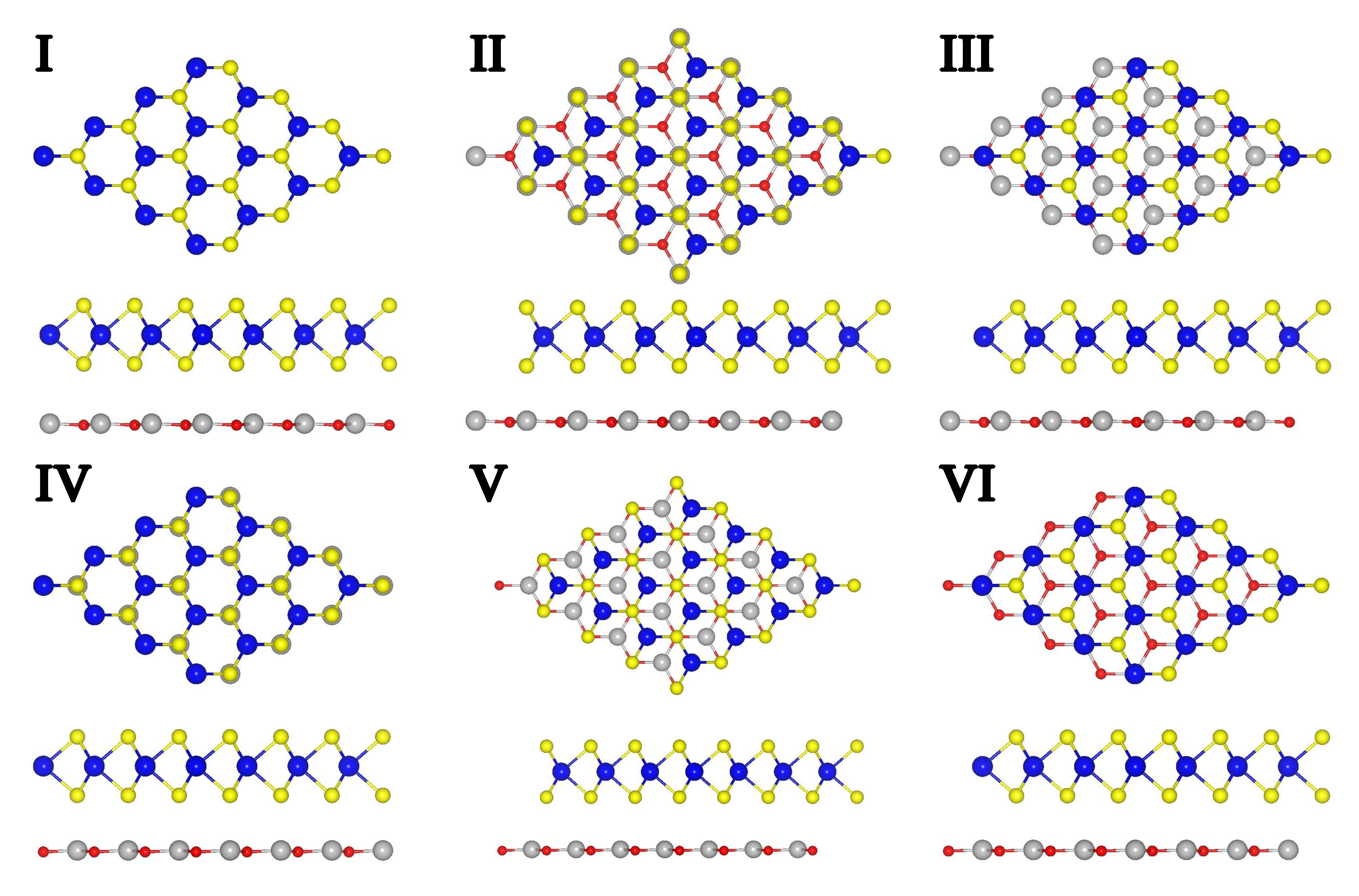


**Fig. S1.** Schematic illustration of different stacking patterns of MX_2_/ZnO heterostructures; the red, yellow, grey, and blue spheres represent M, X, Zn, and O atoms, respectively.
